# A novel method for the histolocalization of Al and Pb in root tissues, tested in *Typha domingensis* Pers. grown in iron mining tailings

**DOI:** 10.1007/s00709-026-02217-7

**Published:** 2026-05-23

**Authors:** Marcelo Ramos de Anchieta, Fabricio José Pereira

**Affiliations:** 1 Instituto de Ciências Naturais (ICN), Universidade Federal de Lavras, Campus Universitário, Lavras, Minas Gerais 37200-900 Brazil; 2 Instituto de Ciências da Natureza (ICN), Universidade Federal de Alfenas, Rua Gabriel Monteiro da Silva, 700, Centro, Alfenas, MG 37130-001 Brazil

**Keywords:** Cattail, Solid wastes, Metal tolerance, Phytoremediation, Lead, Aluminum

## Abstract

The objective of this work was to test a new method for Al and Pb histolocalization using Chrome Azurol S (CAS) and Pyrogallol Red (PGR), respectively, using roots from *Typha domingensis* Pers. plants grown in iron mining tailings. For both CAS and PGR, we tested two solvents (distilled water pH 6.7 and 100 mM phosphate buffer pH 5.0), two concentrations (0.1% and 0.5%, m V^− 1^), and three reaction times (1, 5, and 10 min). We tested CAS and PGR histolocalization of Al and Pb in *T. domingensis* plants, which were grown for 180 days in flooded iron mining tailings containing 2385.6 mg Al Kg^− 1^ and 6.8 mg Pb Kg^− 1^; as a control, we used plants from a natural population from an unpolluted location. *T. domingensis* roots grown in iron mining tailings accumulated 1384 mg Kg^− 1^ of Al and 18 mg Pb Kg^− 1^. CAS and PGR promoted no reaction in tissues from unpolluted roots; however, CAS and PGR reacted with root tissues from polluted roots under all tested conditions. CAS staining evidenced cytosolic Al present in purple and red vesicles in epidermal and cortical root cells from polluted roots. PGR application in polluted roots highlighted the presence of cytosolic Pb in pale-pink and red vesicles in the epidermis and cortex. Both CAS and PGR produced a reddish hue in the cell walls from the vascular cylinder, indicating the presence of Al and Pb. Thus, CAS and PGR can be used for Al and Pb histolocalization in polluted root tissues.

## Introduction

Several regions in the world have been suffering with disasters originated from iron mining tailings dam failures, which were reported in Europe (Rico et al. 2008), Asia (Lin et al. 2022) and Americas (Carmo et al. 2017). In Brazil, a big iron mining tailings’s dam failure ocurred in 2015, releasing about 50 million m^3^, causing a large environmental disaster (Lacaz et al. 2017; Carmo et al. 2017). Works using these tailings showed a high concentration of Al and Pb, which have the potential to cause phytotoxicity (Gomes et al. 2025; da Silva et al. 2024; Caetano et al. 2022). Tolerant species have been identified for use in revegetation and phytoremediation of areas impacted by iron mining tailings relying in photosynthetic, growth and leaf anatomical parameters (Gomes et al. 2025; da Silva et al. 2024; Caetano et al. 2022). Nonetheless, detailed cellular tolerance mechanisms are still unknown for these species being the subcellular allocation of Al and Pb an important aspect of these mechanisms.

*Typha domingensis* Pers. is an aquatic plant found in most regions in the world as an invasive species but which shows potential for remediation systems (Duarte et al. 2021; Sesin et al. 2021), being tolerant to toxic metals such as Cd (de Oliveira et al. 2022), Cr (Mufarrege et al. 2015), Pb (Bonanno et al. 2013), and Al (Hegazy et al. 2011), being these pollutants mostly allocated in the roots of the species. This species was also identified as tolerant to gold mining tailings (Compaore et al. 2020), although no information is found about its tolerance to iron mining tailings. *T. domingensis* is a potential species to use in impacted areas by iron mining tailings, which are waterlogged, such as the areas impacted in the Doce river basin (Lacaz et al. 2017; Carmo et al. 2017). Although the plant is recognized as tolerant to Al and Pb (Hegazy et al. 2011; Bonanno et al. 2013), the cellular allocation mechanisms were never identified for these metals in *T. domingensis* roots.

Al is often found at higher concentrations in cytosolic vesicles as shown for *Nicotina tabacum* L. (Ozturk et al. 2023), *Triticum aestivum* L. (Tice et al. 1992), and *Glycine max* L. (Lazof et al. 1994); however, Al can also be allocated to cell walls in *Eriophorum vaginatum* L. (Kisiala et al. 2021). Similarly, Pb is also allocated to cytoplasmic vesicles (Kopittke et al. 2008; Pandley et al. 2020) and in cell walls (Krzesłowska et al. 2009). No information is found about the cellular allocation of Al and Pb in plants growing in iron mining tailings.

There are available methods for Al and Pb histolocalization, but these methods show low sensitivity and specificity. Because no molecular markers for Al histolocalization are available, most methods for the detection of the element are based on stains (Ozturk et al. 2023). For instance, Al is commonly detected using hematoxylin (Tanoi et al. 2006; Ryan et al. 1993; Singh et al. 2021). This method, however, shows several limitations because hematoxylin reacts easily with aluminum phosphates compared with ionic Al or other forms (Ownby 1993). Hematoxylin also shows a high affinity for iron and can stain several cellular components, such as chromosomes, cytoplasm, cellulose, mitochondria, plastids and the cell nucleus (Johansen 1940). Some fluorescent stains can be used for the detection of Al, an example is morina and lumogalion, a fluorescent molecule used for Al detection, which is more sensitive compared to hematoxylin (Ezaki et al. 1995; Tanoi et al. 2006; Valeur and Berberan-Santos 2011; Kisiala et al. 2021); however, these methods are more expensive and require fluorescent microscopes. Pb histolocalization can be performed using sodium rhodizonate at pH 2.8 (Glater and Hernandez 1972), 4-(2-pyridylazo)resorcinol, and dithizone (Seregin and Ivanov 1997); however, these stains show low specificity to Pb. Thus, developing new methods with higher sensitivity and lower costs for the histolocalization of Al and Pb is advantageous.

Chrome Azurol S (CAS) is a highly sensitive analytical reagent that is used for spectrophotometric determination of Al in solutions (Pakalns 1965). CAS reacts with Al forming purple complexes which can change the color to red after longer times (Molina-Diaz et al. 1993; Ni et al. 2007). The Pyrogallol Red (PGR) is also a reagent used for Pb quantification in solutions using spectrophotometry (Anderson and Brown 1981). PGR reacts with Pb, creating pale-pink or red complexes (Inoue et al. 2017). CAS and PGR are intensely used for Al and Pb quantification analyses with established and reliable protocols; however, these reagents were never tested for the histolocalization of these elements, despite their high sensitivity and colouring capacity being key traits for potential histological stains.

This work hypothesizes that CAS and PGR can be used for the histolocalization of Al and Pb in plant tissues by reacting with these metals, which can be allocated in vesicles or in the cell walls. Thus, the objective of this work was to test the use of CAS and PGR for the histolocalization of Al and Pb in roots from *T. domingensis* grown in iron mining tailings containing these elements.

## Materials and methods

### Stains and experiments

Analytical grade reagents were used for these experiments, with chrome azurol S (CAS) (Sigma-Aldrich) for aluminum (Al) and Pyrogallol Red (PGR) (Sigma-Aldrich) for lead (Pb). Chrome azurol S is a well-known stain used for spectrophotometric determination of Al, being highly sensitive (1 µg mL^− 1^) and can be used at room temperature since it was described by Pakalns (1965). CAS reacts with Al, forming usually colored complexes in liquid or solid phases (Molina-Diaz et al. 1993). The colored complex formed by the CAS and Al reaction usually shows a purple color but shifts to red with time (Ni et al. 2007). PGR reacts with Pb, being used for the quantification of the metal in water and plant tissues, being recognized by its high precision and sensitivity (0.002 µg mL^− 1^) for spectrophotometric applications (Anderson and Brown 1981). The colored complex formed by PGR and Pb reaction shows a pale-pink or red color (Inoue et al. 2017). Despite being used for spectrophotometric and colorimetric analysis, both CAS and PGR were tested for histolocalization of metals for the first time in this work.

Spectrophotometric analyses of CAS and PGR use aqueous solutions at pH 7 or lower (Pakalns 1965; Anderson and Brown 1981). In this work, we performed experiments for different conditions for CAS and PGR application in histolocalization of Al and Pb, being: the concentration of the stain (0.1% or 0.5%), the type of solution (distilled water or phosphate buffer), and the reaction time (1, 5, and 10 min). These experiments were further detailed in this section. For all tests, we used *Typha domingensis* Pers. roots from two conditions: collected from non-polluted sites and from plants grown in iron mining tailings polluted with Al and Pb; these conditions will be further explained in this section.

### Solution type test

Two types of solutions were tested, being CAS and PGR diluted in distilled water or phosphate buffer. Distilled water was selected because it is the main solvent for CAS and PGR for spectrophotometric analysis (Pakalns 1965; Anderson and Brown 1981). The pH value of distilled water was measured using a pH meter (BEL Engineering, Monza, Italy), being 6.7. This is a pH value viable for CAS and PGR reactions, but a lower pH value is more commonly used (Pakalns 1965; Anderson and Brown 1981). In addition, Al and Pb are both more available under pH 5 or lower (Rahman and Upadhyaya 2021; Kumpiene et al. 2008). Thus, we tested the 100 mM phosphate buffer at pH 5 as the solvent for CAS and PGR to provide lower pH levels, possibly favoring the reaction of stains with Al and Pb. For the phosphate buffer preparation, 1.3609 g of potassium phosphate monobasic (KH_2_PO_4_) was weighed in an analytical scale AY 220, precision 0.0001 g (Shimadzu, Tokyo, Japan) and then diluted in 100 mL of distilled water. The pH of the distilled water was 6.7. In addition, 0.8709 g of potassium phosphate dibasic (K_2_HPO_4_) was weighed on an analytical scale and diluted in 50 mL of distilled water. Then, 98.6 mL of the KH_2_PO_4_ solution was mixed with 1.4 mL of the K_2_HPO_4_ solution. The pH value obtained was 5.03, being measured with a pH meter (BEL Engineering, Monza, Italy).

### Stain concentration test

The efficiency of the detection of a given compound in plant tissues (histolocalization) depends on the concentration of the stain, and for most histochemical tests, stain concentrations are found between 0.1 and 1% (m V^− 1^) (Johansen 1940). We tested two concentrations for both CAS and PGR stains, being 0.1 and 0.5% (m V^− 1^). These concentrations are close to those used for other stains in histolocalization (Johansen 1940) as well as similar concentrations used for CAS in spectrophotometric analyses, which is 0.165% (Pakalns 1965) and for PGR, 0.16% (Anderson and Brown 1981). For the preparation of 0.1% and 0.5% solutions, 0.01–0.05 g of CAS or PGR was weighed on an analytical scale and diluted in 10 mL of the solvent. For each stain (CAS and PGR) two concentrations (0.1 and 0.5%) were prepared for each solvent (distilled water or phosphate buffer) totaling eight solutions as follows: CAS 0.1% and 0.5% in distilled water, CAS 0.1% and 0.5% in phosphate buffer, PGR 0.1% and 0.5% in distilled water and PGR 0.1% and 0.5% in phosphate buffer.

### Reaction time test

Histolocalization of compounds in plant tissues also depends on the time of the reaction, which varies from seconds to minutes, depending on which stain and compound is studied (Johansen 1940). We tested 1, 5, and 10 min of reaction time. For this, sections were transferred to CAS and PGR solutions, and after 1, 5, or 10 min of reaction, sections were removed and transferred to distilled water to remove the excess stain, and then mounted on slides containing distilled water and covered with coverslips.

### Plant material

*Typha domingensis* Pers. individuals were collected from a natural population in Alfenas, State of Minas Gerais, Brazil (21º24’06.2” S, 45º59’07.5” W) and were transferred to a greenhouse in the Universidade Federal de Alfenas – MG. These plants were washed in tap water and added to 20 L trays containing nutrient solution (Hoagland and Arnon 1950) for propagation. This population is located in an area without any significant source of Al or Pb. We used the following sources and concentrations for the nutrient solution: NH_4_H_2_PO_4_ (0.4 mM), KNO_3_ (0.6 mM), Ca(NO_3_)_2_ (0.2 mM), Mg(NO_3_)_2_ (0.1 mM), K_2_SO_4_ (0.1 mM), FeSO_4_ 7H_2_O (0.003 mM), H_2_BO_3_ (0.0025 mM), MnSO_4_ H_2_O (0.0002 mM), ZnSO_4_ 7H_2_O (0.0002 mM), CuSO_4_ 5H_2_O (0.00005 mM), H_2_MoO_4_ H_2_O (0.00005 mM) without any source of Al or Pb. Plants were grown in a greenhouse for 60 days to obtain clones, which were used for experiments. Water lost by evapotranspiration was replaced daily, and nutrient solution fortnightly. Greenhouse conditions during this period averaged 25 ± 2 °C, 72.4% relative humidity, and 187 W m^− 2^ of photosynthetically active radiation.

### Iron mining tailings

Iron mining tailings were collected 4.0 Km from the Fundão dam in Mariana, state of Minas Gerais, Brazil (20°11’58"S and 43°29’28” W), at 1 m depth; further, they were transported to the Universidade Federal de Alfenas and stored in plastic bags and in a room protected from humidity and direct radiation until the start of the experiments. The tailings analysis for Al and Pb contents was performed using the protocol from AOAC (2005). Mining tailings were oven-dried at 60 °C until reaching a constant mass and submitted to different extraction methods for elemental quantification. For this, 500 mg of the tailing separated and 50 mL of KCl was applied for Ca, Mg, and Al solubilization, while the K and PO_4_^-^ contents were extracted with 50 mL of 0.025 mol L^− 1^ H_2_SO_4_ + 0.05 mol L^− 1^ HCl. Micronutrients were measured using a chelating solution, and PTEs were extracted after nitroperchloric digestion. The levels Al and Pb were quantified using an atomic absorption spectrophotometer, AAnalyst 800 (PerkinElmer, Waltham, MA, USA) (Abbruzzini et al. 2014). The concentration in tailings was 2385.6 mg Kg^− 1^ for Al and 6.8 mg Kg^− 1^ for Pb.

### *T. domingensis* cultivation in iron mining tailings

Clones acclimated to the greenhouse were selected by similar size (50 cm in height and four leaves, containing rhizomes and roots). A total of 18 clones were transferred to individual pots containing 2 L of sieved iron mining tailings, which were waterlogged until a water layer of 3 cm was formed above the tailings. Iron mining tailings were sieved in a 2.5 mm mesh before being added to the pots. Water availability was maintained by replacing the water lost by evapotranspiration daily until reaching a mark indicating 3 cm of depth. The experiment was conducted in a greenhouse at 25 ± 2 °C, 72.4% relative humidity, and 187 W m^− 2^ for 180 days. At the end of the experiment, plants were removed from tailings, washed in tap water, and three roots per plant (54 roots) were collected and fixed in 70% (V V^− 1^) ethanol for the histolocalization tests, and the remaining were oven-dried at 40 °C to obtain the dry mass for Al and Pb quantification.

### Al and Pb quantification in *T. domingensis* roots

Al and Pb quantification was performed according to AOAC (2005). For this analysis, 500 mg of plant material was oven-dried at 60 °C for 72 h and then transferred to tubes containing 6 ml of HNO_3_ and kept at 25 °C overnight. Tubes were then placed in a digestion block and heated to 140 °C. Samples were kept at this temperature until the volume of the solution was reduced to 1 mL. Samples were cooled, and then 2 mL of HClO_4_ was added, and the solution was heated to 190 °C for 2 h (Abbruzzini et al. 2014). Samples were then cooled, and the quantification of Al and Pb was performed in an atomic absorption spectrometer AAnalyst 800 (PerkinElmer, Waltham, MA, USA). Root Al content was 1384 mg Kg^− 1^ and Pb was 18 mg Kg^− 1^.

### Unpolluted roots

In order to test the stains in unpolluted roots, *T. domingensis* plants were collected from natural populations located in Alfenas, state of Minas Gerais, Brazil (21°25’37"S 45°56’15"W) in a site without any source of potential Al or Pb contamination. Roots and rhizomes were collected at the site, transported to Universidade Federal de Alfenas, and washed in tap water. The entire roots were fixed in 70% ethanol (V V^− 1^), a total of 18 roots were collected for the histolocalization tests and served as a control.

### Preparation of microscopy slides

Roots fixed and previously collected from the unpolluted and polluted plants were used for staining tests. Root sections were performed using steel blades 2 cm away from the root cap and collected in distilled water. Histolocalization of chemical compounds in plant tissues depends on the quality of the section (Johansen 1940), and clarification can help with the method by removing pigments and other compounds that can mislead the result. For clarification, we applied 50% sodium hypochlorite (V V^− 1^) prior to the application of the stains. For this solution, 50 mL of sodium hypochlorite (NaClO) solution containing 20 g L^− 1^ of NaClO was diluted with 50 mL of distilled water, giving a final concentration of 10 g L^− 1^ of NaClO. Sections were exposed to the NaClO solution for 10 min and washed twice in distilled water prior to application of CAS and PGR. Sections were then submitted to tests described for the CAS and PGR, being mounted in slides. Three slides were made per root. Slides were observed and photographed in a Nikon Eclipse Si microscope (Nikon, Tokyo, Japan).

## Results

### Histolocalization of Al

**Fig. 1:**
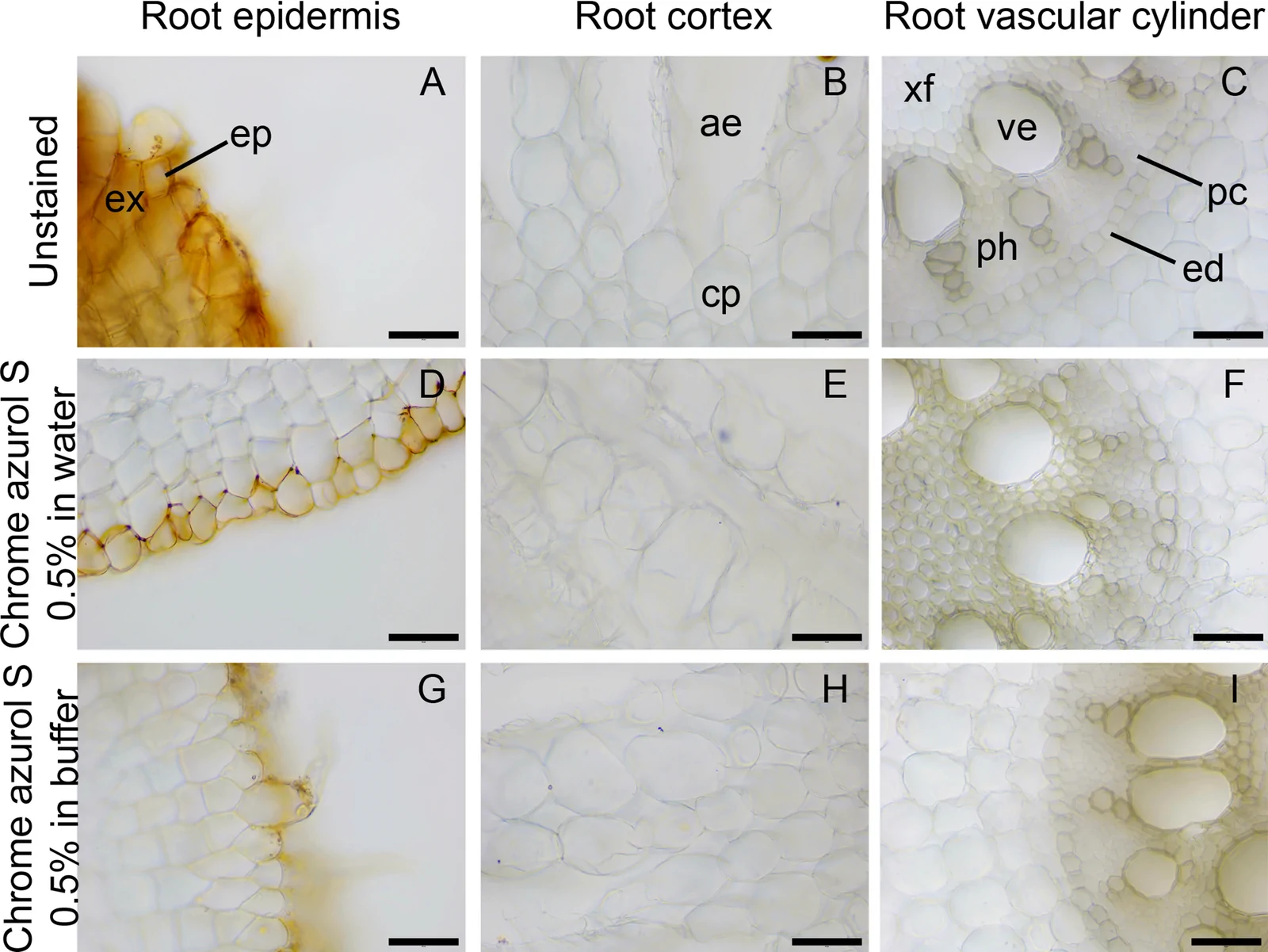
Transversal sections from unpolluted roots of *T. domingensis*. **A**, **B**, and **C**: sections unstained with chrome azurol S (CAS), **D**, **E**, and **F**: sections stained with CAS 0.5% in water with 10 min of reaction, **G**, **H**, and **I**: sections stained with CAS 0.5% in phosphate buffer with 10 min of reaction time. ep = epidermis; ex = exodermis; ae = aerenchyma; cp = cortical parenchyma; ed = endodermis; pc = pericycle; ph = phloem; ve = vessel element; xf = xylem fibers. Bars: 50 μm

**Fig. 2:**
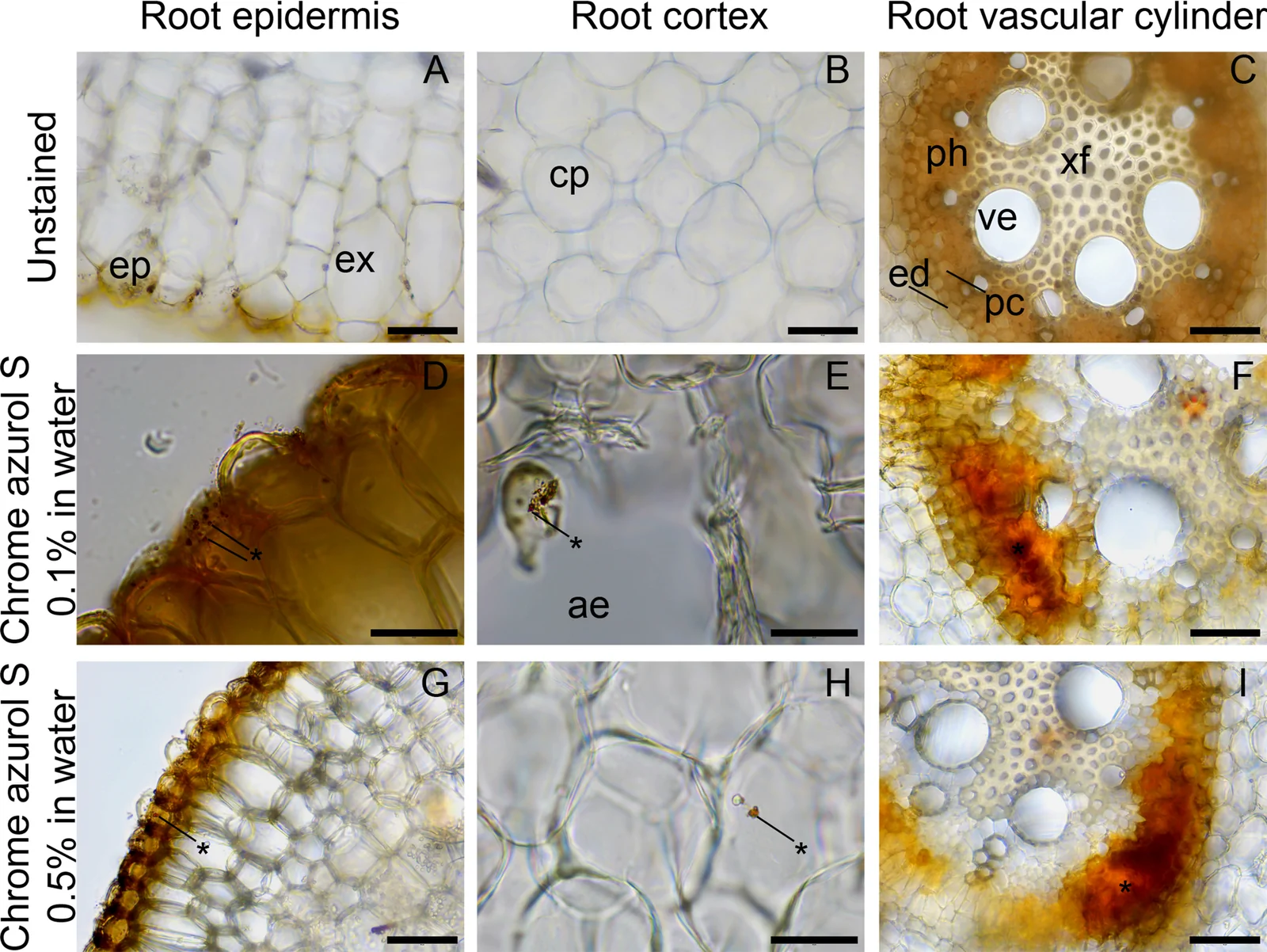
Transversal sections from roots of *T. domingensis* grown in iron mining tailings containing Al (2385.6 mg Kg^−1^_tailings_), these roots accumulated 1384 mg Al Kg^−1^_root dry mass_. **A**, **B**, and **C**: sections unstained with chrome azurol S (CAS), **D**, **E**, and **F**: sections stained with CAS 0.1% in distilled water with 5 min of reaction, **G**, **H**, and **I**: sections stained with CAS 0.5% in distilled water with 5 min of reaction time. ep = epidermis; ex = exodermis; ae = aerenchyma; cp = cortical parenchyma; ed = endodermis; pc = pericycle; ph = phloem; ve = vessel element; xf = xylem fibers. * = example of stained cell vesicle or cell walls containing Al. Bars: **A**, **B**, **C**, **F**, **G**, and I = 50 μm; **D**, **E**, and H = 25 μm

**Fig. 3:**
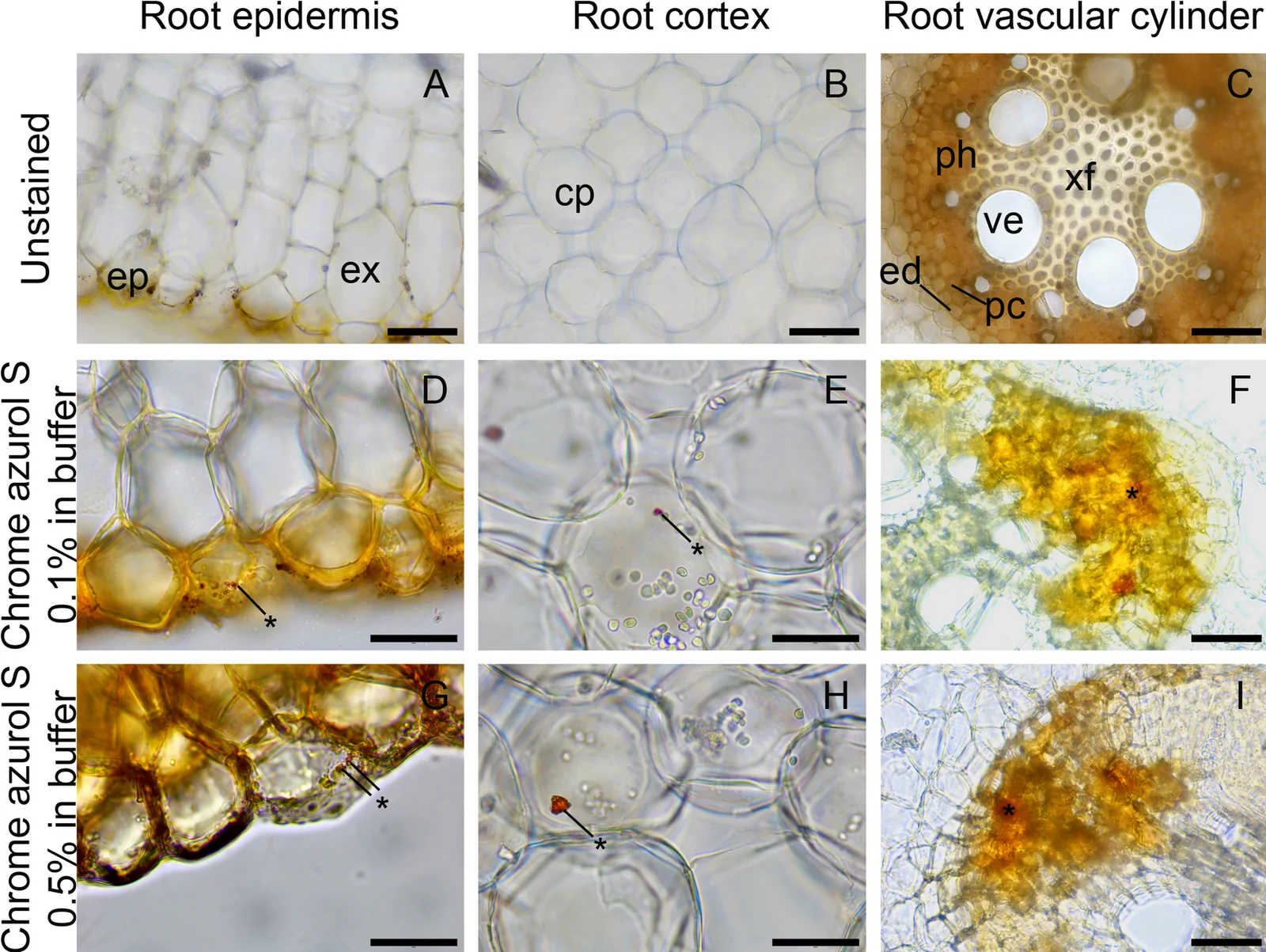
Transversal sections from roots of *T. domingensis* grown in iron mining tailings containing Al (2385.6 mg Kg^−1^_tailings_), these roots accumulated 1384 mg Al Kg^−1^_root dry mass_. **A**, **B**, and **C**: sections unstained with chrome azurol S (CAS), **D**, **E**, and **F**: sections stained with CAS 0.1% in 100 mM phosphate buffer, pH 5, with 5 min of reaction, **G**, **H**, and **I**: sections stained with CAS 0.5% in phosphate buffer with 5 min of reaction time. ep = epidermis; ex = exodermis; ae = aerenchyma; cp = cortical parenchyma; ed = endodermis; pc = pericycle; ph = phloem; ve = vessel element; xf = xylem fibers. * = example of stained cell vesicle or cell walls containing Al. Bars: **A**, **B**, **C**, **F**, and I = 50 μm; **D**, **E**, **G**, and H = 25 μm

**Fig. 4:**
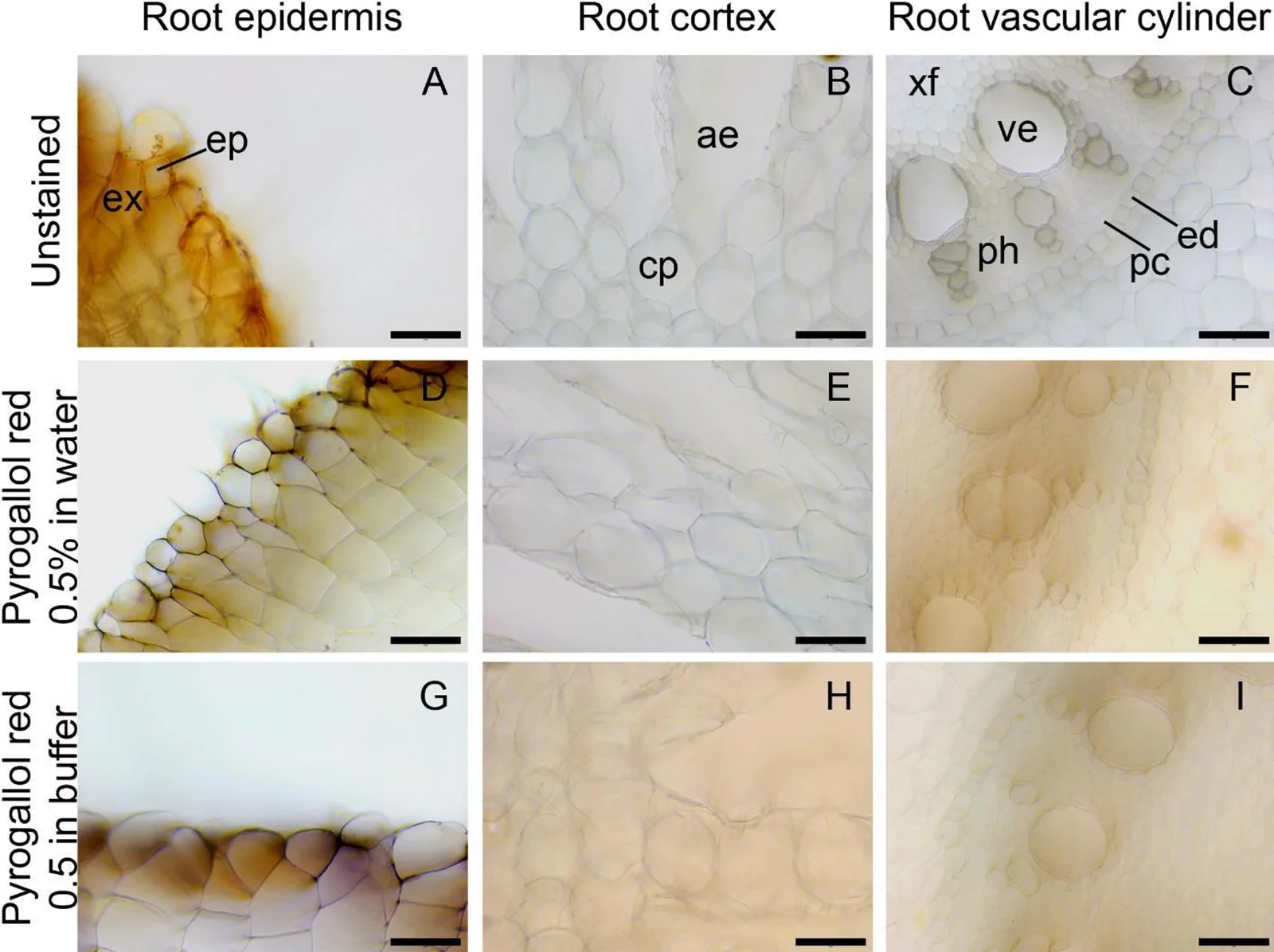
Transversal sections from unpolluted roots of *T. domingensis*. **A**, **B**, and **C**: sections unstained with pyrogallol red (PGR), **D**, **E**, and **F**: sections stained with PGR 0.5% in water with 10 min of reaction, **G**, **H**, and **I**: sections stained with PGR 0.5% in phosphate buffer with 10 min of reaction time. ep = epidermis; ex = exodermis; ae = aerenchyma; cp = cortical parenchyma; ed = endodermis; pc = pericycle; ph = phloem; ve = vessel element; xf = xylem fibers. Bars: 50 μm

**Fig. 5:**
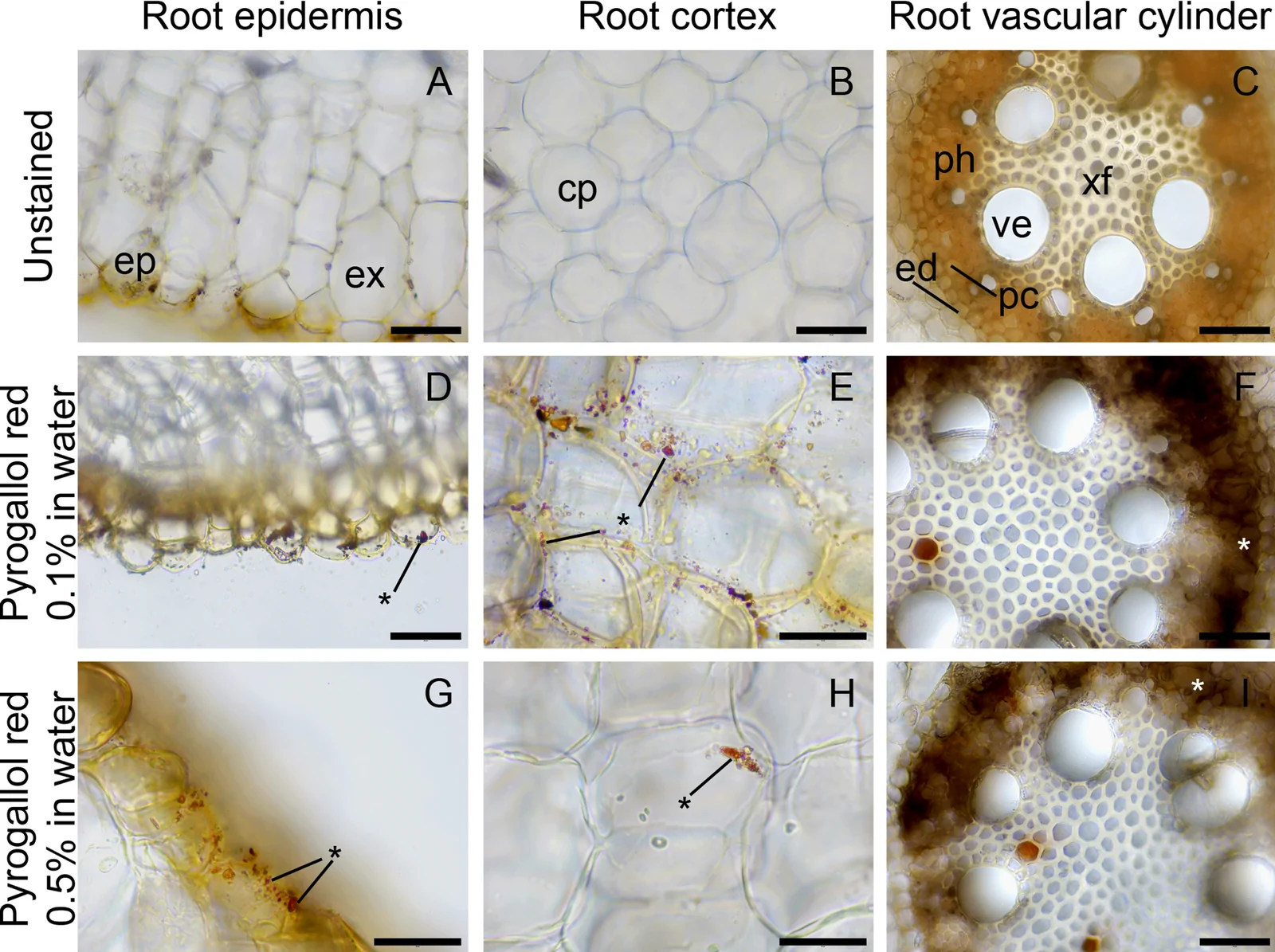
Transversal sections from roots of *T. domingensis* grown in iron mining tailings containing Pb (6.8 mg Kg^−1^_tailings_), these roots accumulated 18 mg Pb Kg^−1^_root dry mass_. **A**, **B**, and **C**: sections unstained with pyrogallol red (PGR), **D**, **E**, and **F**: sections stained with PGR 0.1% in distilled water with 5 min of reaction, **G**, **H**, and **I**: sections stained with PGR 0.5% in distilled water with 5 min of reaction time. ep = epidermis; ex = exodermis; ae = aerenchyma; cp = cortical parenchyma; ed = endodermis; pc = pericycle; ph = phloem; ve = vessel element; xf = xylem fibers. * = example of stained cell vesicle or cell walls containing Pb. Bars: **A**, **B**, **C**, **D**, **F** e I = 50 μm; **E**, **G** e H = 25 μm

**Fig. 6:**
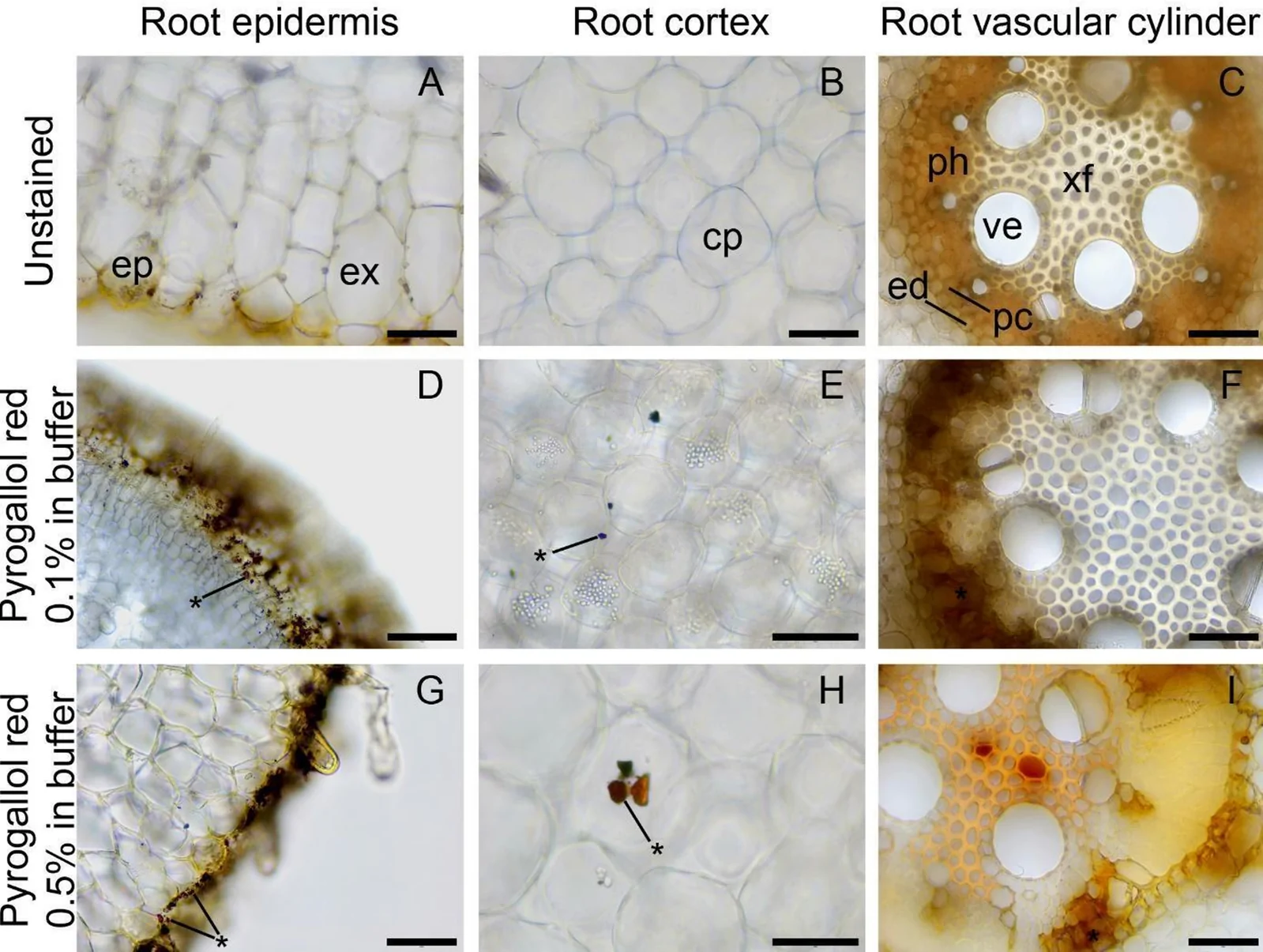
Transversal sections from roots of *T. domingensis* grown in iron mining tailings containing Pb (6.8 mg Kg^−1^_tailings_), these roots accumulated 18 mg Pb Kg^−1^_root dry mass_. **A**, **B**, and **C**: sections unstained with pyrogallol red (PGR), **D**, **E**, and **F**: sections stained with PGR 0.1% in phosphate 100 mM phosphate buffer pH 5 with 5 min of reaction, **G**, **H**, and **I**: sections stained with PGR 0.5% in phosphate buffer with 5 min of reaction time. ep = epidermis; ex = exodermis; ae = aerenchyma; cp = cortical parenchyma; ed = endodermis; pc = pericycle; ph = phloem; ve = vessel element; xf = xylem fibers. * = example of stained cell vesicle or cell walls containing Pb. Bars: **A**, **B**, **C**, **D**, **E**, **F**, **G** e I = 50 μm; H = 25 μm

It is important to understand the root anatomy of *T. domingensis* prior to describing the effectiveness of histolocalization of Al and Pb. This root can be separated into three main regions: epidermis, cortex, and vascular cylinder. The epidermis is formed by one layer of elongated cells at the outermost part of the root; these cells are alive and show no intercellular spaces. This region is shown in Figs. 1, 2, 3, 4, 5 and 6 in panels A, D, and G. In the center of the root transversal sections, it is located the vascular cylinder which contains the pericycle, xylem, and phloem. This region is shown in Figs. 1, 2, 3, 4, 5 and 6 in panels C, F, and I. The cortex region is located between the epidermis and vascular cylinder, and it is formed by several tissues, mainly the exodermis (more externally and adjacent to the epidermis), the aerenchyma (central, forming several large chambers), and the endodermis (one layer in the innermost part of the cortex). This region is shown in Figs. 1, 2, 3, 4, 5 and 6 in panels B, E, and H. All these tissues contain living cells, except for the xylem fibers shown at the center of the vascular cylinder.

The Fig. 1 shows the results of the application of chrome azurol S (CAS) in unpolluted roots. The Fig. 1A and B, and 1C exhibit sections without the application of CAS and show no evidence of deformations or Al deposits. Comparing these images with Fig. 1D and I, which show the application of CAS at the highest concentration tested (0.5%) and at the longer reaction time (10 min) and showed no reactions using water (Fig. 1D and E, and 1F) or phosphate buffer as solvent (Fig. 1G and H, and 1I).

The unstained root sections from *T. domingensis* plants grown in iron mining tailings showed no significant deformations or accumulation evidence in their tissues (Fig. 1A, B, and C). Nonetheless, these iron mining tailings showed significant Al concentration, and these roots accumulated high Al levels (Fig. 1). The application of aqueous chrome azurol S (CAS) in root sections effectively stained tissues, highlighting Al-accumulation sites (Fig. 2D-F). CAS detected the presence of Al in red-colored and purple spherical vesicles present in the root epidermal and cortical cells (Fig. 2D, E, G, and H). In the vascular cylinder, these vesicles are not visible, but the cell walls of the phloem and xylem showed a reddish color, differing from unstained tissues, which exhibit cell walls with a natural brown-orange stain (Fig. 2F and I). This reddish stain from CAS also indicates that Al is present in the xylem and phloem cell walls. However, the concentration of CAS did not affect the stain histolocalization (Fig. 2D-I). The reaction time also did not influence the histolocalization, because CAS effectively stained vesicles and cell walls containing Al in all tested times (1, 5, and 10 min). Figure 2 shows sections stained for 5 min.

The application of chrome azurol S (CAS) diluted in 100 mM phosphate buffer, pH 5, promoted the same results compared with distilled water (Fig. 3). Unstained sections of *T. domingensis* roots showed no detectable vesicles or hued cell walls in tissues (Fig. 3A, B, and C). Application of CAS in sections evidenced the presence of red and purple vesicles containing Al in the epidermal and cortical cells (Fig. 3D, E, G, and H), and cell walls of xylem and phloem showed a redish stain (Fig. 3F and I). Neither the concentration of CAS (Fig. 3) nor the reaction time affected the results, since sections stained equally in all concentrations and reaction times tested. Figure 3 shows images from sections stained for 5 min.

### Histolocalization of Pb

The application of pyrogallol red (PGR) in unpolluted roots promoted no staining of vesicles or cell walls and root tissues look indistinguishable from unstained sections (Fig. 4). PGR solutions in both distilled water or 100 mM phosphate buffer as solvents caused the same results with the absence of stained tissues evidencing the absence of significant Pb accumulation in unpolluted roots (Fig. 4).

Polluted roots from *T. domingensis* plants grown in iron mining tailings accumulated significant Pb levels (Fig. 5). Unstained sections of these roots, despite accumulating Pb, showed no evidence of the presence of the metal (Fig. 5A, B, and C). Nonetheless, sections stained with pyrogallol red (PGR) in distilled water evidenced pale-pink or red vesicles located in epidermal and cortical root cells (Fig. 5D, E, G, and H) as well as a redish color in the cell walls of xylem and phloem cells in the vascular cylinder (Fig. 5F and I), indicating the presence of Pb. Both PGR concentrations (0.1% and 0.5%) stained the Pb location sites equally (Fig. 5), and all reaction times tested also promoted the same effects. the Fig. 5 shows images from sections stained for 5 min.

The application of pyrogallol red (PGR) using 100 mM phosphate buffer, pH 5, promoted similar effects compared to solutions using distilled water. PGR evidenced the presence of Pb in the epidermal and cortical cells with pale-pink or red vesicles (Fig. 6D, E, G, and H) and a redish color in the cell walls of xylem and phloem cells (Fig. 6F and I). The PGR concentrations tested (0.1% and 0.5%) and the reaction times (1, 5, or 10 min) promoted indistinguishable effects. Figure 6 shows both concentrations and reaction times of 5 min.

## Discussion

### Al histolocalization using CAS

Results from this work support the use of CAS for Al detection in plant tissues. CAS application on polluted roots revealed stained vesicles and cell walls, which are absent in non-polluted samples, supporting the accumulation of Al in root tissues from polluted roots. These results suggest that the use of CAS for Al detection in plant tissues is a reliable method because the location and type of tissues where these are expected for Al compartmentalization match those reported in previous works, and the color patterns match those predicted for CAS reactions in solutions.

The color of vesicles and cell walls evidenced in polluted roots from *T. domingensis* roots stained with CAS is similar to the reaction of this stain with Al in solution. The CAS reaction with Al in aqueous solutions forms complexes with different colors, with blue complexes being the most common (Molina-Diaz et al. 1993); but purple complexes are also reported (Ni et al. 2007). Nonetheless, longer reaction times change the color of CAS complexes to red (Ni et al. 2007; Sasirekha and Srividya 2016). Vesicles observed in tissues of polluted roots from *T. domingensis* were mostly red, but some still showed purple hue (Figs. 2 and 3). Because blue and purple Al-CAS complexes are quickly formed in solution (Molina-Diaz et al. 1993; Ni et al. 2007) and the color of the complex can change to red after some minutes (Ni et al. 2007; Sasirekha and Srividya 2016); it is possible that the time tested in this work for the use of CAS in histolocalization (1 min or longer) favored the red staining of vesicles and also promoted a reddish coloration to cell walls. Thus, both colors visualized for vesicles (purple and red) are in accordance with reported Al-CAS complexes, supporting the effectiveness of CAS for histolocalization of Al in plant tissues; however, the reddish color of cell walls suggests a weaker reaction.

Al can be accumulated in plant tissues mainly in vesicles in the cytosol, but a smaller amount can also be deposited in cell walls; moreover, the allocation depends on plant species and organs. The use of a fluorescent stain in the roots of *Nicotina tabacum* L. suggests that Al accumulates mainly in the cytosol (Ozturk et al. 2023). In addition, Tice et al. (1992) reported that between 60 and 70% of the Al uptaken by *Triticum aestivum* L. roots remain in the cytoplasm or cell nucleus. Similar results can also be found for soybean (*Glycine max* L.) that accumulates Al in the cytosol (Lazof et al. 1994). Al can not be stored in the cytosol without some compartmentalization, which is obtained by the vesicles. This supports our results suggesting a higher intracellular (cytosolic) Al accumulation in polluted roots of *T. domingensis*; being this the first work to access this subject with this species, there is no previous information to compare.

Results also suggest that the reddish color of cell walls (Figs. 2 and 3) is related to a weaker reaction, likely because there is less Al accumulated in cell walls. It is also possible that Al in cell walls forms complexes with other compounds being not available to react with CAS. Al can be accumulated in cell walls from different plant species, such as *Avena sativa* L. (Marienfeld and Stelzer et al. 1993) and *Eriophorum vaginatum* L. (Kisiala et al. 2021). This is the first work showing results indicating the cellular sites of Al accumulation in *T. domingensis*, and results suggest that the main site for Al accumulation is in cytosolic vesicles, which can be evidenced with CAS staining.

### Pb histolocalization using PGR

Results support the capacity of PGR for detecting Pb in plant tissues. PGR evidenced pale-pink and red vesicles in tissues from polluted roots of *T. domingnesis*, which match the color formed by Pb-PGR complexes in solution. According to Inoue et al. (2017), Pb forms red or pale-pink complexes with PGR in aqueous solutions. In addition to these vesicles, cell walls in the vascular cylinder also showed a reddish color in polluted roots of *T. domingensis*, indicating a weaker reaction. The evidenced vesicles and reddish coloration in cell walls were not visible in unpolluted roots, further corroborating the capacity of PGR for Pb detection in plant tissues and that this metal is mainly accumulated in vesicles in *T. domingensis* roots.

Pb is mainly uptaken and accumulated in plant roots (Lyubenova et al. 2012). Pb accumulation in roots is related to apoplastic barriers such as the exodermis and endodermis, which retain the pollutant in roots, avoiding damage to the photosynthetic shoot (Ribeiro et al. 2025). The Pb uptake can react with the pectins from cell walls of root cells, immobilizing this pollutant in roots (Krzesłowska et al. 2009). This causes plants to usually accumulate Pb in roots, as shown for *Salix babylonica* L. (Xue et al. 2020), *Fagopyrum tataricum* (L.) Gaertn. (Wang et al. 2020) and *Zea mays* L. (Malone et al. 1974). There are no works describing the Pb allocation in *T. domingensis*, but the species can accumulate much higher levels of Cd in roots compared to the shoot (de Oliveira et al. 2018). The Pb accumulation in vesicles is a common trait for several plant species, such as * Urochloa decumbens*(Stapf) R. D. Webster and *Chloris gayana* Kunth (Kopittke et al. 2008), and *Cymbopogon flexuosus* (Nees ) Will. Watson (Pandley et al. 2020). There are no works showing the intracellular mechanism of metal accumulation in *T. domingensis*, but results from this work suggest that Pb is accumulated mainly in the cytoplasmic vesicles, evidenced by reaction with PGR.

### Tested parameters for the histolocalization of Al and Pb

Results showed that both CAS and PGR are viable as stains for histolocalization of Al and Pb respectivelly. Interestingly, several parameters tested worked without relevant variation. This is important because it emphasize that CAS reaction with Al and PGR with Pb are strong enough to form complexes in plant tissues as well as in aqueous solutions. Thus, the lowest concentration tested (0.1%) and the lowest reaction time (1 min) worked in both solutions (distilled water and phosphate buffer pH 5). The CAS concentration of 0.1% is similar to that used in solutions for colorimetric detection of Al as shown in Pakalns (1965). The PGR concentration used for colorimetric detection is also close to 0.1% (Anderson and Brown 1981). Thus, it is expected that 0.1% for both CAS and PGR would be sufficient to react with Al and Pb even in plant tissues. Both CAS and PGR solutions for detecting Al and Pb are prepared with lower pH values (5 or lower) (Rahman and Upadhyaya 2021; Kumpiene et al. 2008). However, for histolocalization of Al and Pb in plant tissues reactions were independent of the solutions with different pH tested which turns as favorable result because it can be easier to prepare the reagent. In addition, the shortest time used (1 min) was sufficient for the reaction, which is also relevant for the efficiency of the method. Histolocalization of compounds depends on the concentration of the stain and the reaction time (Johansen 1940). Thus, both CAS and PGR histolocalization of Al and Pb are both high efficient and can be performed at the lowest concentration tested 0.1% at the shortest time (1 min) and using both distilled water or phosphate buffer.

## Conclusion

Chrome Azurol S (CAS) is capable of Al histolocalization in plant tissues and can be used at 0.1% (m V^− 1^) or higher concentrations using distilled water or phosphate buffer 100 mM pH 5.0 and at least 1 min of reaction time. Reaction of CAS with Al form red or purple Al-CAS complexes evidenced in cytoplasmic vesicles and promote a reddish hue in cell walls containing Al.

Pyrogalol Red (PGR) is capable of Pb histolocalization in plant tissues and can be used at 0.1% (m V^− 1^) or higher concentrations using distilled water or phosphate buffer 100 mM pH 5.0 and at least 1 min of reaction time. Reaction of PGR with Pb form pale-pink or red Pb-PGR complexes evidenced in cytoplasmic vesicles and promote a reddish hue in cell walls containing Pb.

Roots of *Typha domingensis* plants grown in flooded iron mining tailings seem to accumulate Al and Pb mainly in vesicles in the cytosol of epidermal and cortical cells.

## Data Availability

No datasets were generated or analysed during the current study.
